# Celiac disease with cerebral and peripheral nerve involvement mimicking multiple sclerosis

**Published:** 2014-09-25

**Authors:** J Finsterer, F Leutmezer

**Affiliations:** * Krankenanstalt Rudolfstiftung, Vienna, Austria; ** Department of Neurology, Medical University Vienna, Austria

**Keywords:** immunology, autoantibodies, immunosuppression, interferon, nerve conduction

## Abstract

Abstract

Objectives: Due to the similarity in the clinical presentation, morphology, and course, celiac disease (CD) may be mixed up with other immunological disorders, such as multiple sclerosis (MS).

Case report: In a 43-year-old Caucasian male with a history of diarrhea and colics since young age, progressive sensory disturbances developed since age 18 years. At age 34, he was diagnosed as relapsing-remitting MS upon an inflammatory CSF-syndrome and non-specific white matter lesions and treated with interferon beta-1b during the next 8 years without effect. At age 35, axonal polyneuropathy and ataxia were diagnosed. Despite normal anti-gliadin, endomysial, and transglutaminase antibodies, CD was diagnosed at age 41, based upon the history, polyneuropathy, positivity for HLA-DQ2 and HLA-DQ8, the white matter lesions, and a beneficial response of the gastrointestinal problems and polyneuropathy to gluten-free diet.

Conclusions: CD may mimic MS and may be present despite the absence of anti-gliadin, endomysial or transglutaminase antibodies. CD should be considered if there is a gastrointestinal problem, polyneuropathy, and ataxia, even if CSF and MRI findings are suggestive of MS.

## Introduction

Celiac disease (CD) is a chronic autoimmune disorder that affects up to 1% of the population in the Western world [**1**]. CD results from an immunological response, characterized by villous atrophy and crypt hyperplasia in the small intestine after exposure to gluten or related proteins found in wheat, rye, or barley [**2**]. Though CD is predominated by gastrointestinal manifestations, it is, in the majority of the cases, a multisystem disease [**3**]. Other affected organs besides the gastrointestinal tract include the cerebrum, muscle, peripheral nerves, endocrine organs, heart, blood cells, or the integument (**Table 1**) [4,5]. Due to the similarity in the clinical presentation, morphology, and course, CD may be mixed up with other immunological disorders, such as multiple sclerosis (MS), as in the following case. 

**Table 1:** Multiorgan manifestations of CD

CNS	White matter lesions Inflammatory CSF-syndrome Sinus venous thrombosis Epilepsy Cerebellar-pontine atrophy Encephalopathy Myelopathy Dementia Gluten ataxia Headache, migraine Depression/anxiety Cerebellar ataxia
Peripheral nervous system	Axonal polyneuropathy Myopathy Polymyositis Dermatomyositis Inclusion-body-myositis Neutrophilic myositis Hypokalemic rhabdomyolysis, tetany Osteomalacic myopathy Hyper-CK-emia
Cardiovascular	Takayashu disease Dilated cardiomyopathy Myocarditis
Gastrointestinal	Dental enamel disease Recurrent aphthous ulcers Vitamin-D deficiency Malabsorption Short stature Autoimmune hepatitis Hepatopathy Primary biliary cirrhosis Nonspecific hepatitis Primary sclerosing cholangitis Nonalcoholic fatty liver disease
Endocramium	Diabetes Hashimoto thyroiditis
Kidneys	Nephrolithiasis
Blood cells	Iron-deficiency anemia Leucopenia Thrombocytopenia
Bones, joints	Osteoporosis Osteomalacia Arthritis
Integument	Dermatitis herpetiformis Vitiligo

## Case report

The patient is a 43-year-old Caucasian male, with a history of diarrhea since age three months, when diet with wheat and milk was begun. Diarrhea did not stop before changing to vegetables and potatoes. At age 9, abdominal colics occurred until late puberty. Since then diarrhea or colics did not recur but episodes of unformed faeces occurred. In 1986, he noted that he frequently lost his slippers and experienced straddling of the toes when stretching his legs. He did no longer tolerate wearing shoes because of hyperalgesia and allodynia and took them off whenever possible. In 2002, the diagnostic work up revealed an inflammatory CSF-syndrome (17/3 cells, 84mg/dl protein, 1.2mg/dl intrathecal IgG, positive oligoclonal bands) and multiple white matter lesions on MRI, which is the reason why he was diagnosed as relapsing-remitting MS with an EDSS score of 1.0. Interferon beta-1b was started and given during the next 8 years without a significant effect or evident side effects. Neurological exam in 5/2003 showed reduced tendon reflexes on the lower limbs, slight ataxia, and stocking-type pallhypesthesia. Nerve biopsy revealed a burned-out, axonal polyneuropathy. Sarcoidosis was excluded by a normal angiotensin converting enzyme-level and negative whole body gallium scintigraphy. Cerebral white matter lesions were unchanged in 2005 except for the regression of the hyperintensity in the left cerebellar peduncle. Cerebral MRI in 2008 revealed a new lesion in the left thalamus. Since 2009 nightly muscle cramps in the calves occurred. In 2010, the diagnostic work-up revealed normal anti-gliadin antibodies (**Table 2**) but positivity for HLA-DQ2 and HLA-DQ8 (genotype C/T, presence of alleles HLA DQA1*0501, *0505, HLA DQB1*0201, *0202, *0302 by means of a SSP-PCR) [**6**]. Cerebral MRI showed a white matter lesion in the left parietal region and the left cerebellar peduncle. In 2/2010, the patient decided to follow a strict gluten-free diet, which resulted in a marked improvement of the gastrointestinal abnormalities but hardly affected the gluten ataxia. In 7/2011, osteoporosis was diagnosed.

**Table 2:**
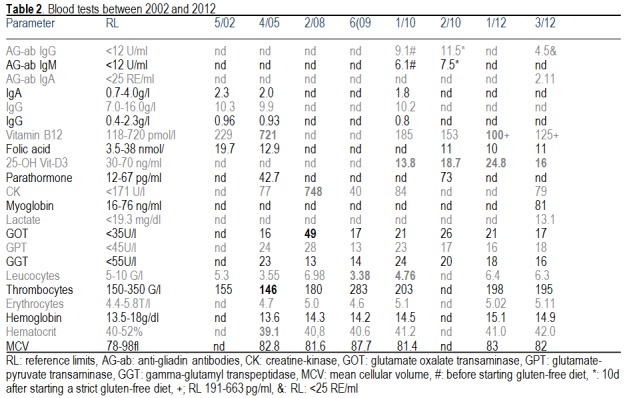
Blood tests between 2002 and 2012

 At a follow-up in 4/2012, he admitted to have drunk alcohol excessively between 1985 and 1995 and to be impotent for some time. Neurological exam revealed gaze-evoked nystagmus, brady diadochokinesis, intention ataxia on the left side, stocking type hypoesthesia on the lower limbs, absent tendon reflexes on the lower limbs, and ataxic stance and gait, this is why he used two crutches for walking. Standing without support resulted in a tendency to fall. Blood tests revealed elevated myoglobin, vitamin-B12 deficiency, and vitamin-D-deficiency, but no gliadin (endomysial) and transglutaminase autoantibodies were found (**Table 2**). Nerve conduction studies revealed a slight improvement compared to previous investigations, such that the sural nerve could be stimulated again and that nerve conduction velocity of the right peroneal nerve improved (**Table 3**). 

**Table 3:**
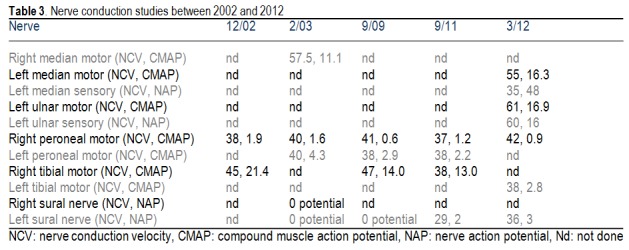
Nerve conduction studies between 2002 and 2012

 Compared to 2010, cerebral MRI showed an old subcortical, frontotemporal, band-like hyperintensity and an old hyperintensity in the left cerebellar peduncle, and a new left parietal, paramedian hyperintensity, a new hyperintense, spot-like periventricular lesion on the right side, and a microadenoma of the pituitary gland. The gastroenterologists refused biopsy of the gastric or colonic mucosa, since he was on a gluten-free diet for 2 years and since only one third of CD patients with gluten ataxia have evidence of enteropathy on biopsy [**2**]. In 4/2012, his medication comprised ibandrone acid every 3 months exclusively. He was still on a strict gluten-free diet. 

## Discussion

 This case is interesting for the mimicry of MS with CD and the diagnosis of CD in the absence of gliadin, endomysial, and transglutaminase antibodies. CD was diagnosed upon the clinical presentation with typical gastrointestinal abnormalities starting in early infancy [**7**], polyneuropathy [**8**], progressive ataxia [**2**], and instrumental findings, such as dynamic white matter lesions [**9**], nerve conduction studies [**10**], densitometry [**11**], a positive status for HLA-DQ2 and HLA-DQ8, and the beneficial response of some CD manifestations to gluten-free diet [**7**]. Further results of blood tests, which supported the diagnosis of CD in the presented patient, included reduced serum levels of vitamin-B12 and vitamin-D. Anti-gliadin antibodies were repeatedly negative. Mucosal biopsy was not carried out, since its sensitivity was regarded as being low after 2 years of gluten-free diet. Differential diagnoses such as Whipple disease, HIV, X-linked gammaglobulinemia, IgA-deficiency, hypo-gammaglobulinemia, cystic fibrosis, or hyper-IgM syndrome were excluded upon clinical, blood chemical, serological, and imaging techniques. 

 CD in the presented patient was misinterpreted as MS because of the CSF and MRI findings. CSF investigations showed a typical constellation usually seen in MS. Visually-evoked potentials were normal. Since MRI showed non-specific white matter lesions, MS was diagnosed and an appropriate treatment initiated. Chronic gastro-intestinal abnormalities and polyneuropathy were attributed to previous alcohol consumption. Since CD occurs in up to 11% of the patients, together with MS [**16-14**], it cannot also be excluded that the described patient simultaneously suffered from CD and MS. Arguments for the coexistence of both conditions are the CSF and MR findings, improvement of gastrointestinal manifestations and polyneuropathy upon gluten-free diet, and previous reports about the collateral occurrence of both conditions [**14**]. Arguments against a coexistence of both conditions, however, are that interferon did not improve the condition during the 8th years period, the clinical presentation could be exclusively explained by CD, the frequency of CD is not increased in MS patients [**15**], gastro-intestinal complaints were the initial manifestations [**8**], and MRI findings could be explained by a number of disorders other than MS. Elevated CSF protein, pleocytosis and positive oligoclonal bands have been previously reported in CD patients [**16,17**]. Whether interferon beta-1b over 8 years has deteriorated CD, remains speculative, but a negative effect cannot be completely excluded since interferon-beta 1b may induce CD [**18**] and since interferon may unmask CD [**19**]. 

 Absence of anti-gliadin antibodies is not unusual and has been reported in a small portion of CD patients [20,21]. Various causes can be put forward to explain the absence of anti-gliadin antibodies in the presented patient. One of the major reasons may be the limited sensitivity/specificity of the antibody tests [**22,23**]. Sensitivity is particularly different between assays for antibodies against native gliadin and tests for antibodies to deamidated gliadin [**23**]. A further reason for antibody-negative CD may be a negative sero-conversion of antibodies during the disease course [**24**]. A third reason for the absence of anti-gliadin antibodies may be the long-standing immunosuppressive treatment with interferon [**25**]. The absence of endomysial and transglutaminase antibodies may be due to the same reasons as the absence of anti-gliadin antibodies and does not exclude CD, but their presence is a more reliable indicator for CD than anti-gliadin antibodies [**26**]. Since 30% of the general population carries one of the CD-associated HLA alleles and only 3% of the individuals with one or both alleles develop CD, presence of HLA-DQ2/DQ8 is not diagnostic, although their absence essentially excludes CD [**27**].

 In conclusion, this case shows that CD may mimic MS and that CD may be present despite the absence of anti-gliadin antibodies. If the history is indicative of a gastro-intestinal problem, CD should be considered even if CSF and MRI findings are suggestive of MS. An inflammatory CSF-syndrome is not unique to MS. Since clinical patterns of CD vary considerably, presentation can pose a diagnostic challenge. Clinicians of all disciplines should keep CD in mind when evaluating patients and clinical education to diagnose CD should be improved not to overlook the disease for many years.
